# A database of synthetic inelastic neutron scattering spectra from molecules and crystals

**DOI:** 10.1038/s41597-022-01926-x

**Published:** 2023-01-24

**Authors:** Yongqiang Cheng, Matthew B. Stone, Anibal J. Ramirez-Cuesta

**Affiliations:** Neutron Scattering Division, Oak Ridge National Laboratory, Oak Ridge, Tennessee 37831 USA

**Keywords:** Atomistic models, Cheminformatics, Structure of solids and liquids

## Abstract

Inelastic neutron scattering (INS) is a powerful tool to study the vibrational dynamics in a material. The analysis and interpretation of the INS spectra, however, are often nontrivial. Unlike diffraction, for which one can quickly calculate the scattering pattern from the structure, the calculation of INS spectra from the structure involves multiple steps requiring significant experience and computational resources. To overcome this barrier, a database of INS spectra consisting of commonly seen materials will be a valuable reference, and it will also lay the foundation of advanced data-driven analysis and interpretation of INS spectra. Here we report such a database compiled for over 20,000 organic molecules and over 10,000 inorganic crystals. The INS spectra are obtained from a streamlined workflow, and the synthetic INS spectra are also verified by available experimental data. The database is expected to greatly facilitate INS data analysis, and it can also enable the utilization of advanced analytics such as data mining and machine learning.

Notice: This manuscript has been authored by UT-Battelle, LLC under Contract No. DE-AC05-00OR22725 with the U.S. Department of Energy. The United States Government retains and the publisher, by accepting the article for publication, acknowledges that the United States Government retains a non-exclusive, paid-up, irrevocable, world-wide license to publish or reproduce the published form of this manuscript, or allow others to do so, for United States Government purposes. The Department of Energy will provide public access to these results of federally sponsored research in accordance with the DOE Public Access Plan (http://energy.gov/downloads/doe-public-access-plan).

## Background & Summary

Neutrons, like X-rays, can be used to measure the atomic-level structure and dynamics in a material^1,2^. Compared to X-ray scattering, neutron scattering has some unique advantages, making it a very useful complementary tool to provide a complete picture of where atoms are and what they do^3^. Thermal neutrons used in most neutron scattering experiments have energy and momentum comparable to phonons, the vibrational quanta in materials, so that the vibrational dynamics can be measured with high accuracy and resolution using inelastic neutron scattering (INS). Thanks to the high neutron scattering cross-sections of light elements, neutrons are very sensitive to H, C, O, etc., which are sometimes difficult to see with X-rays especially when in the presence of heavier elements. Unlike Raman or Infrared spectroscopy, INS does not suffer from selection rules, meaning that all phonons can in principle contribute to the total scattering intensity, making it an ideal technique to measure phonon dispersion and phonon density of states (PDOS). Neutrons are also highly penetrating, so that the measured data reflect the statistical results of the bulk sample. It also means more complex and intrusive sample environment can be tolerated. Neutrons also have a magnetic moment allowing them to couple to magnetic moments in materials. This makes them especially useful for studying magnetic structures and magnetic excitations. In actual INS experiments, the magnetic excitations will very often overlap significantly in momentum and energy transfer with the phonon spectrum. Neutron scattering has been used to study materials with fundamental and practical interest in a large variety of areas covering condensed matter physics, chemistry, biology, geology, and engineering^4–6^.

When a neutron beam interacts with a material, the neutrons can be scattered elastically or inelastically^1^. One is able to determine long range ordered structures using diffraction measurements which integrate over all energy transfers, both elastic and inelastic scattering, similar to X-ray diffraction. The data analysis is essentially a refinement process involving calculating the diffraction pattern from a candidate structural model, comparing with experiment, adjusting/optimizing the structural model, and repeating until numerical convergence criteria are achieved. This procedure works because the diffraction pattern due to the long-range order can be analytically and quickly calculated from the structure. In the analysis of INS, however, a similar protocol would be extremely difficult to implement, because there is in general no easy way to obtain the INS spectrum directly from the structure. A typical procedure involves structural optimization and then calculation of vibrational modes, often from density functional theory (DFT) or other first-principles methods^5^. The entire process can take hours or days depending on the complexity of the structure, thus making an iterative process computationally restrictive. Such calculations also require special expertise and significant computing resources (hardware and software). Simulations of INS spectra from the vibrational modes are also more complicated than calculating the diffraction and only recently become more accessible to general users with the development of additional software tools such as the OCLIMAX program^7,8^.

The lack of a venue to quickly “predict” the INS spectra from a structure model, even by a crude approximation, has been a major hurdle in the analysis and interpretation of INS spectra. Users need this capability when they are writing a proposal, planning for an experiment, making decisions during the experiment, or performing analysis after the experiment. Even individuals interested in magnetic scattering can make use of this capability in order to assist in distinguishing magnetic from vibrational scattering channels. To this end, having a database containing the INS spectra of commonly seen materials will be extremely useful. However, due to the extremely limited resources for neutron scattering, an experimental database is expensive and time-consuming to compile. The currently available ISIS database^9^ and DCS database^10^ contain 837 and 11 spectra, respectively. In this work, we have created a large-scale synthetic database containing simulated INS spectra for 20,000+ organic molecules and 10,000+ inorganic crystals. It is made publicly available for use and download for research and development purposes. The database can be used as a reference to search for the target or similar samples of interest, or as training datasets for more advanced analysis using data mining or machine learning.

## Methods

**Fig. 1:**
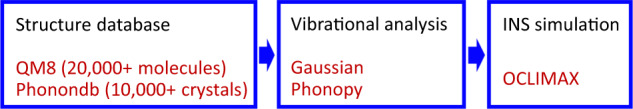
Workflow used to produce the INS spectra database. The QM8 database was used for organic molecules, and the Phonondb was used for inorganic crystals. The vibrational analysis was performed using Gaussian 09 and Phonopy codes, and the resulting INS simulation was produced using OCLIMAX software.

**Fig. 2:**
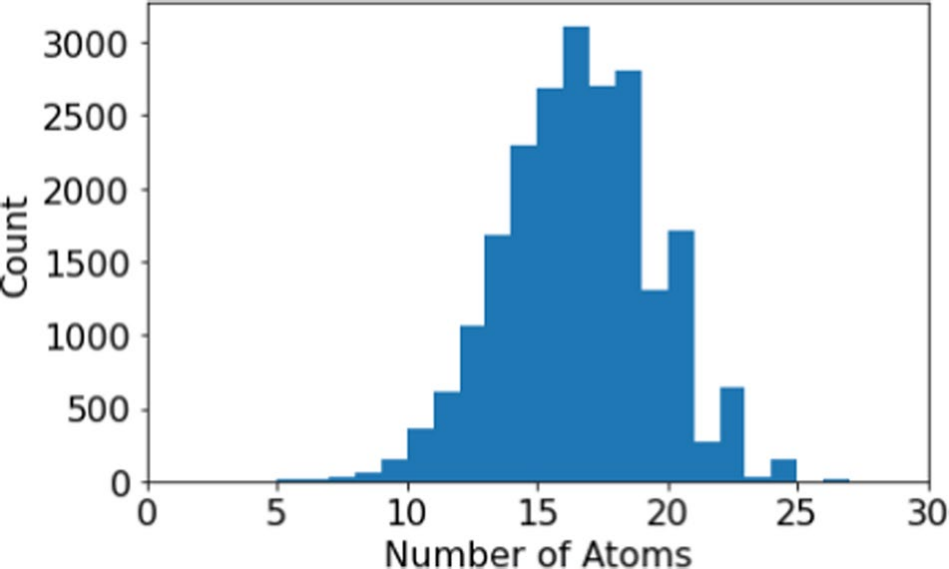
Distribution of number of molecules as a function of molecular size (number of atoms, including hydrogen) in the QM8 database.

The INS spectra for organic molecules and inorganic crystals are produced following the workflow shown in Fig. 1. Specifically, for organic molecules, the molecules in QM8 database^11–13^ were used (molecules containing 8 or fewer non-hydrogen atoms). The distribution of molecular size in this database can be found in Fig. 2. With the starting molecular structure, potential energy minimization (optimization) and vibrational analysis were performed using Gaussian 09^14^, with the following accuracy and key parameters: B3LYP/6-311++G(d,p) OPT = (Tight) Int = (Grid = Ultrafine) CPHF = (Grid = Fine). There are 21,786 molecules in the QM8 database, but 93 failed to reach convergence in the Gaussian calculation. The results for 21,693 molecules are then converted to INS spectra for VISION/TOSCA using OCLIMAX. VISION^15^ and TOSCA^16^ are two indirect geometry neutron spectrometers at the Spallation Neutron Source and ISIS, respectively. They are optimized to measure hydrogen containing materials with a focus on applications in chemistry. The calculated spectra for these instruments can be produced using the default parameters in OCLIMAX software, with an energy unit of cm^−1^ (commonly used in chemical spectroscopy). Also produced from OCLIMAX is an xyz file containing the normal modes, as well as the calculated INS intensity for each individual mode. This allows one to quickly determine the underlying physical origin of features in the calculated spectrum.

**Fig. 3:**
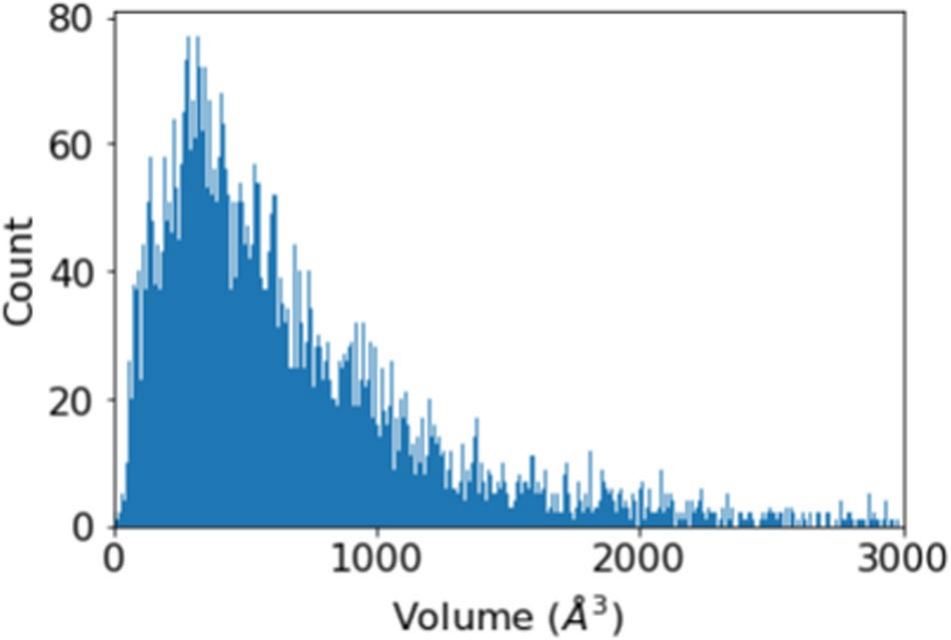
Distribution of number of crystal structures as a function of unit cell volume in the inorganic crystal (phonondb) database. The bin size is 5 Å^3^. Note that there are a small number of unit cells with volume larger than 3000 Å^3^ not shown in this figure.

**Fig. 4:**
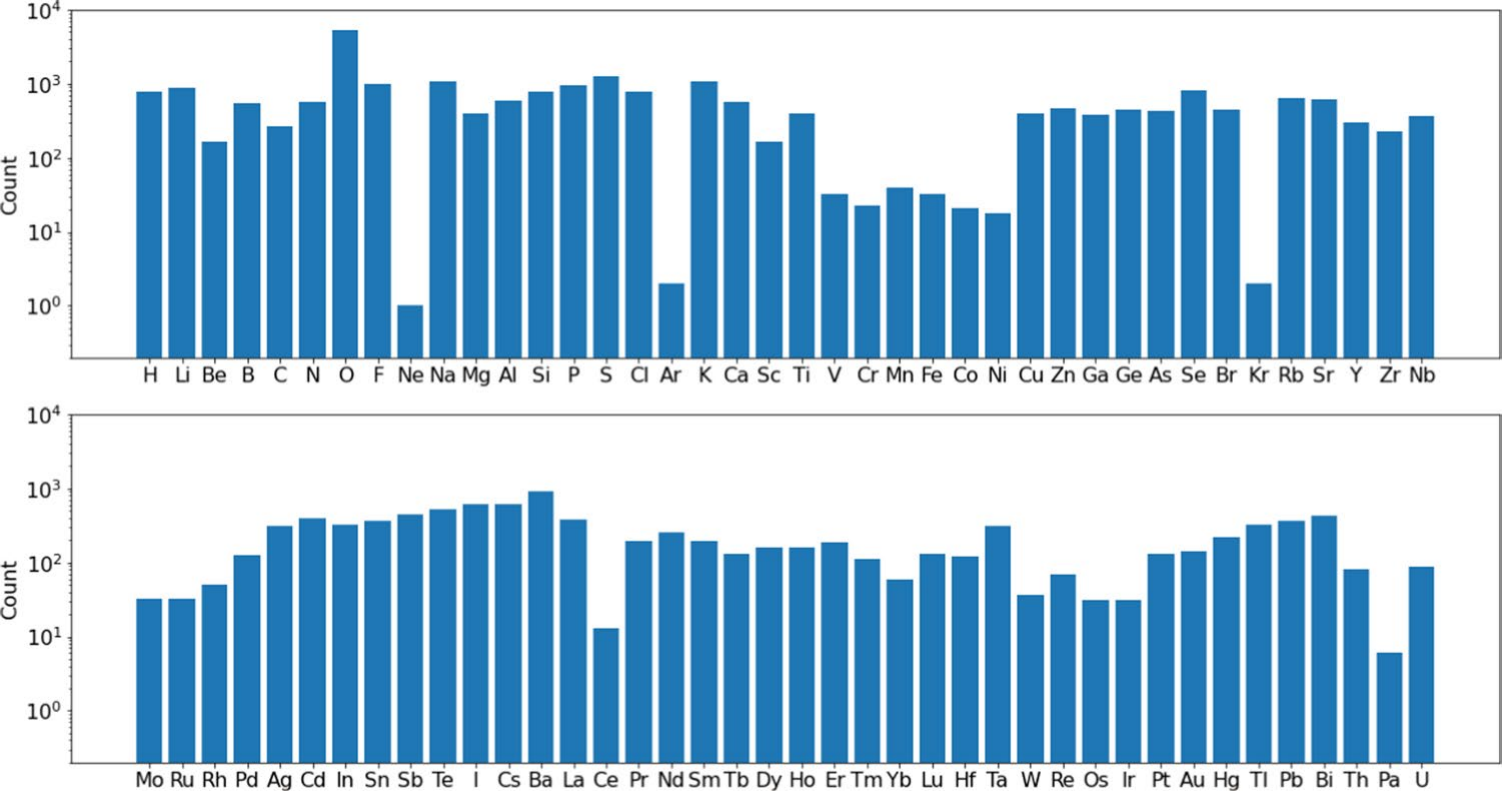
Histogram of the occurrence of each element in the inorganic crystal database (phonondb). Note that the y-axis is in logarithmic scale.

For inorganic crystals, we chose the phonondb@kyoto-u created by Togo^17–19^ as our starting point because unlike some other PDOS databases, this one includes force constants for each crystal calculated from DFT (VASP^20^). This allows us to run customized phonon calculations using Phonopy^21^ to obtain the needed eigenvalues and eigenvectors within the full Brillouin zone for INS simulations. Some statistical information on the 10,032 crystals in this database is shown in Figs. 3,4 and Supplementary Table 1. Note that due to the incomplete neutron scattering cross-section information of Xe^22^, nine Xe-containing compounds were not calculated for INS. The 2D S(Q,E) spectra for the 10,023 inorganic crystals were calculated in the energy range of 0–150 meV (bin size 0.5 meV, 1 meV = 8.066 cm^−1^), Q range of 0–15 Å^−1^ (bin size 0.05 Å^−1^), using an energy resolution of 1.5 meV. The calculations were performed on a computer cluster with 1600 CPU cores. It took about a day of computing time in total, meaning that it would not be an issue to regenerate this database for a different instrument, sample environment, E/Q range, or temperature range. In addition to the 2D S(Q,E), VISION/TOSCA spectra, PDOS and elemental-specific partial PDOS, as well as the corresponding neutron-weighted PDOS, are also calculated.

## Data Records

**Fig. 5:**
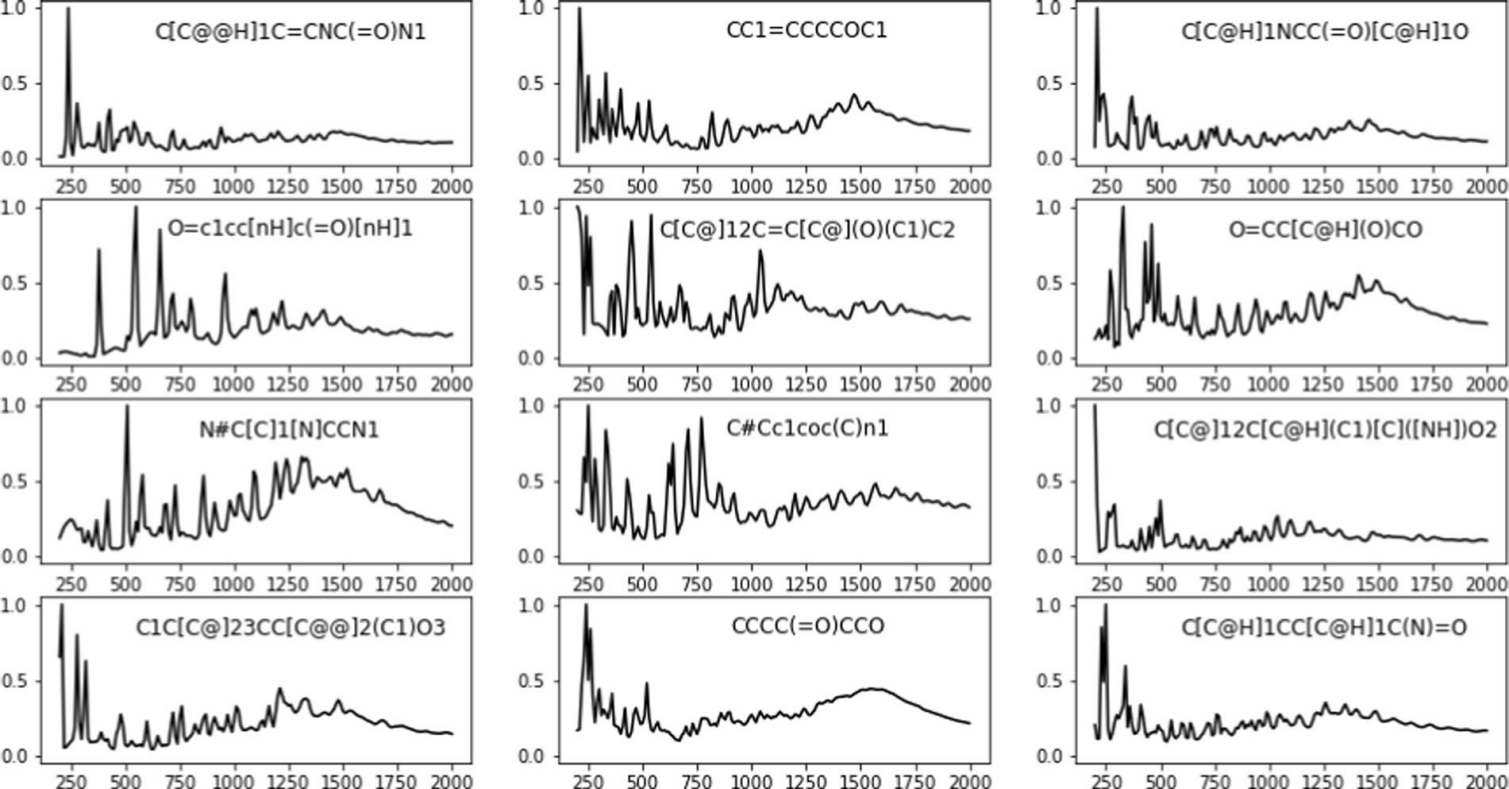
Examples of simulated INS spectra for organic molecules. The legend shows the SMILES^24^ representation of each molecule. The x-axis is frequency in units of wavenumber (cm^−1^) and the y-axis is the calculated normalized scattering intensity. Intensity is normalized such that the strongest peak has a unitary value. The spectra shown here are the total spectra including all contributions from fundamental excitations, combinations, overtones, and phonon wings.

**Fig. 6:**
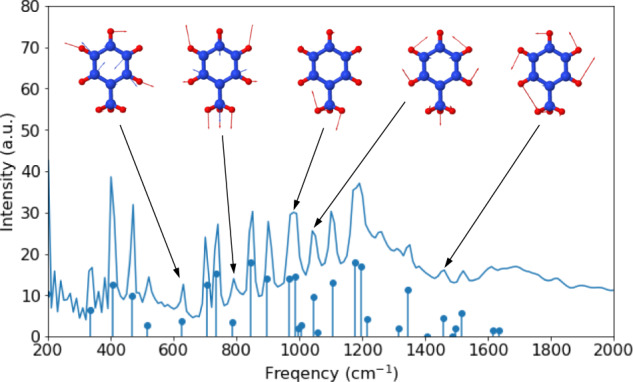
Assignment of toluene vibrational modes to the INS peaks, using the mode.csv file and xyz file provided in the database.

All data files are available at Zenodo^23^. Data files for each structure model are included in a subfolder. For the QM8 molecular database, there are five files in each subfolder: an INFO-* file containing the SMILES^24^ string as well as the IUPAC name (if available) for the particular molecule, a *.com file containing the input for the Gaussian simulation (which also contains the atomic coordinates), a *vis_inc_0K.csv file containing the simulated INS spectra, a *.xyz file containing the displacement of each vibrational modes (can be visualized with Jmol^25^), and a *modes.csv file containing the calculated INS intensity for each normal mode. Some examples of the simulated INS spectra are shown in Fig. 5. When the simulated INS spectrum and the intensity of each mode are plotted together, as shown in Fig. 6, it becomes clear that which mode is responsible for the INS peak observed in the total spectrum. The corresponding normal modes can then be visualized using the xyz file.

**Fig. 7:**
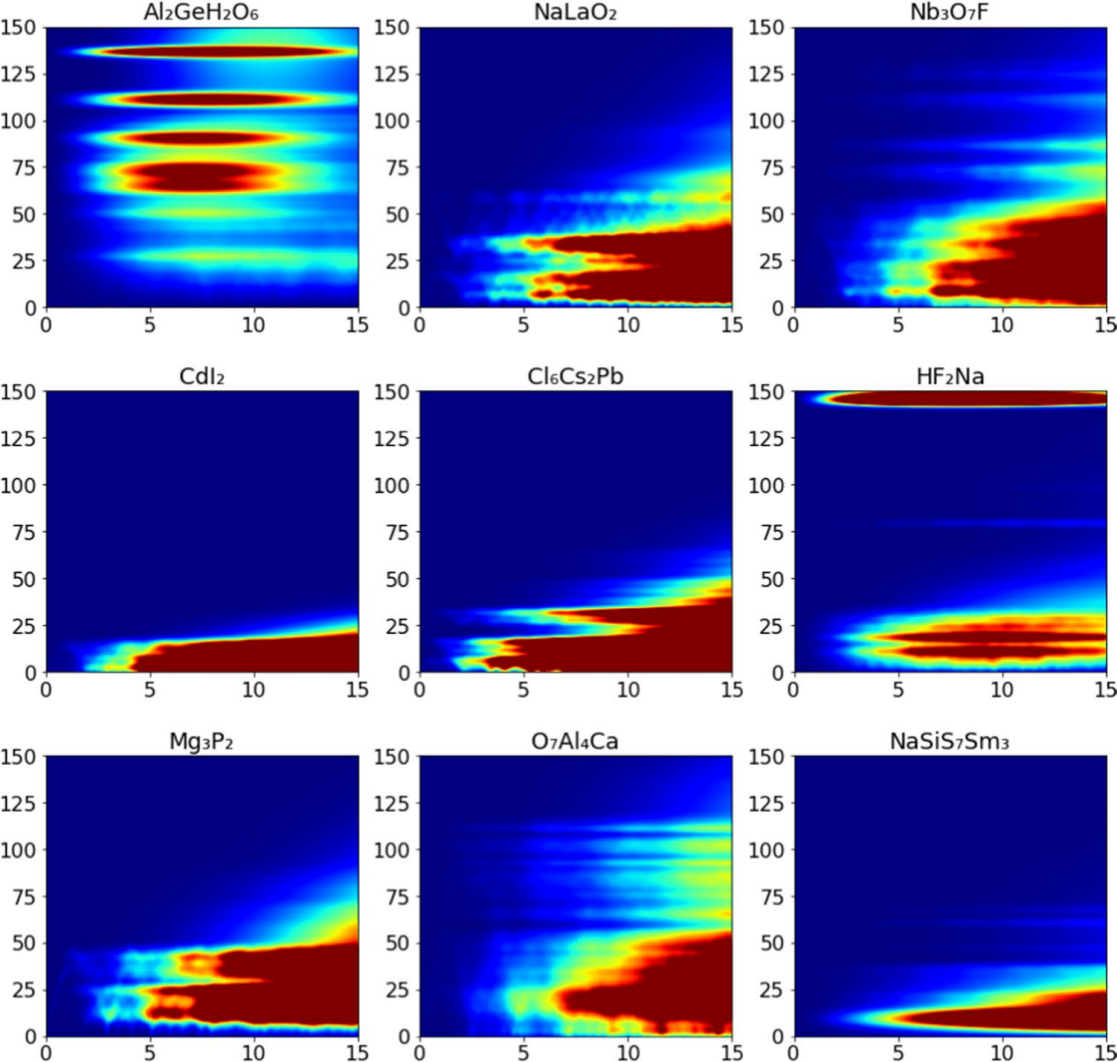
Examples of simulated 2D INS spectra, or S(Q,E), for inorganic crystals. The x-axis is momentum transfer in units of Å^−1^ and the y-axis is energy transfer in units of meV. This view of the calculated spectrum is similar to what would be measured by direct geometry neutron chopper spectrometers^29^.

For the inorganic crystal database, each subfolder also contains five files: a structure.cif file for the crystal structure, a vis_inc_0K.csv file containing the simulated VISION/TOSCA spectra, a powder_2Dmesh_coh_0K.csv file containing the simulated powder S(Q,E), a vis_nwdos.csv file containing the neutron weighted PDOS, a vis_dos.csv file containing the true PDOS, and a gamma_modes.xyz file containing the displacements of gamma point phonons for visualization. Note that the INS spectra and PDOS are calculated on phonons sampled in the full Brillouin zone; the gamma point phonon files are provided for visualization only and not used for INS or PDOS calculations. Examples of the simulated 2D S(Q,E) are shown in Fig. 7.

## Technical Validation

**Fig. 8:**
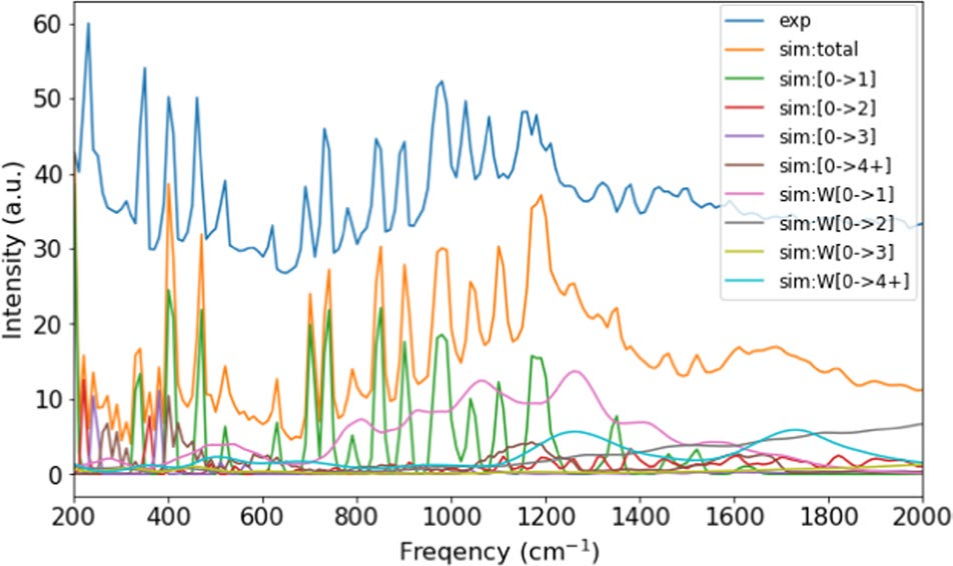
Comparison of the simulated and experimental INS spectra for toluene. The simulated partial spectra are also shown, corresponding to contributions from each level of excitations. For example, [0- > 1] means fundamental excitations. [0- > 2] means two-phonon excitations. W[0- > 1] refers to the phonon wing from fundamental excitations, etc. The experimental spectrum is offset vertically for clarity of the figure.

Although the procedure shown in Fig. 1 to produce the simulated INS spectra hasn’t been previously used on large scale dataset in a high-throughput fashion, it has a proven record to be generally reliable and accurate, as reported in literature (e.g., publications that used OCLIMAX^7^ for INS simulations). Here we provide further validation by directly comparing spectra from the database with experimental data. For molecular systems, the comparison is made for toluene (C_7_H_8_), as shown in Fig. 8. The simulated total spectrum agrees very well with the experimental spectrum collected at VISION. All major peaks can be unambiguously assigned. Furthermore, the simulated partial spectra clearly tell us which peaks are from fundamental excitations, which peaks are from combinations and overtones, and which part of the intensity comes mainly from phonon wings (intermolecular modes in the solid state). It should be noted that in an INS experiment, one measures a solid sample, not a single molecule. In the INS simulations for molecules, the intermolecular interactions are approximately accounted for by the wing calculation^5^. This simplification seems to work well for relatively large molecules with weak intermolecular interactions (such as toluene in Fig. 8), but tends to fail for very small molecules (those containing only a few atoms) with strong intermolecular interactions (such as water ice, ammonia, or methane). Fortunately, experimental data for these very “simple” molecular systems are usually available^9^.

**Fig. 9:**
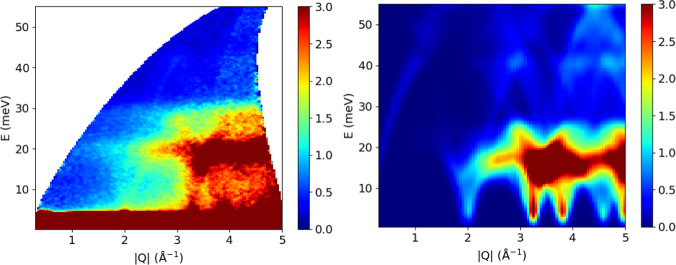
Comparison of experimental (left) and simulated (right) S(Q,E) for silicon powder. The experimental spectrum was collected at SEQUOIA with an incident energy of 60 meV. The simulated spectrum is taken from the database which was obtained with the generic parameters not specifically optimized for the experimental conditions and resolution (which may explain some of the discrepancies). The experimental spectrum also contains contribution from the aluminium sample holder, which has relatively high intensity at around 20 meV and 4 Å^−1^.

Figure 9 illustrates a comparison of 2D S(Q,E) for silicon powder. While the simulation was done with generic parameters as described above and defined in the params file, and not specifically for the instrument setup at SEQUOIA^26^ used to collect the experimental data, the agreement is still satisfactory. Note that there is a small contribution from the aluminium sample container in the experimental spectra, as described in the figure caption.

## Usage Notes

Two tables listing all entries in the database are provided, which can help users to quickly find/locate the files they need. A Python script to plot data is also provided. The INS data files are all in ASCII format and can be imported in text editors and other software packages for post-processing. They can also be loaded in Mantid^27^ and DAVE^28^, which are often used to analyse and visualize INS data.

## Supplementary information

Table 1

## Data Availability

Gaussian 09^14^, Phonopy^21^, and OCLIMAX^7^ are used to generate the datasets. Gaussian 09 is commercial software that requires the users to purchase a license. Phonopy and OCLIMAX are freely available to the public. All parameters used in the calculations are provided in the database as input files.
