# Supplementary material for: A database of synthetic inelastic neutron scattering spectra from molecules and crystals
Source: Sci Data. 2023 Jan 24;10:54. doi: 10.1038/s41597-022-01926-x (PMC9873913; doi:10.1038/s41597-022-01926-x)
Supplement: Supplementary file 1 — Table 1 [file 41597_2022_1926_MOESM1_ESM.pdf]

Supplementary Information

A database of synthetic inelastic neutron scattering spectra from molecules and crystals

Yongqiang Cheng, Matthew B. Stone, Anibal J. Ramirez-Cuesta

Neutron Scattering Division, Oak Ridge National Laboratory, Oak Ridge, Tennessee 37831 USA

Supplementary Table 1. Number of crystals in the phonondb[1] database as a function of space group symmetry.

Symbol	No.	Count	Symbol	No.	Count	Symbol	No.	Count	Symbol	No.	Count
P2_1/c	14	1066	I-43m	217	50	Pmna	53	13	Im-3	204	5
Pnma	62	845	C2	5	46	P-6	174	12	Cccm	66	5
C2/c	15	601	Pc	7	45	Ima2	46	12	P4mm	99	5
C2/m	12	373	I4/m	87	45	P4/nbm	125	12	P4_1	76	5
Fm-3m	225	335	P4/mbm	127	45	I2_13	199	12	Pn-3m	224	5
R-3m	166	317	Ia-3d	230	45	P3_221	154	11	Fd-3	203	5
Cmcm	63	311	Immm	71	45	P2	3	11	P3_112	151	4
R-3	148	197	R3	146	45	P2_12_12	18	11	P3	143	4
P-3m1	164	197	P321	150	42	Pccn	56	11	P-4b2	117	4
P2_1/m	11	192	Pmmn	59	42	P6_222	180	11	P6/mcc	192	4
P6_3/mmc	194	182	P-42_1m	113	40	P4/ncc	130	10	P-4n2	118	4
P3m1	156	170	R3c	161	39	P4_32_12	96	10	P4/nnc	126	4
P4/nmm	129	156	Ia-3	206	37	I-43d	220	10	P3_2	145	4
I4/mmm	139	156	I4_1/acd	142	35	I4mm	107	10	P-4c2	116	3
R-3c	167	154	P3_121	152	35	P4_2/m	84	10	Cmm2	35	3
Fd-3m	227	143	Pnna	52	35	Cmme	67	9	Pmm2	25	3
Pbca	61	129	Ama2	40	35	P-4m2	115	9	P4_2/nbc	133	3
Pna2_1	33	125	P-62m	189	34	P6_322	182	9	P4/mcc	124	3
Pbcn	60	120	C222_1	20	33	P-6m2	187	9	I4_1cd	110	3
I-42d	122	116	P6_3cm	185	32	P4_2/ncm	138	8	P6_122	178	3
P6_3mc	186	115	P-31c	163	31	Pcca	54	8	Pba2	32	3
P2_12_12_1	19	114	Fdd2	43	31	P4_332	212	8	P4_2/mmc	131	3
I4_1/amd	141	106	P-3	147	30	P3_1	144	8	F-43c	219	2
Cmc2_1	36	106	P4_12_12	92	30	P4_2/mcm	132	8	P-42c	112	2
P6_3/m	176	100	Pca2_1	29	29	Pm	6	8	I4	79	2
I4/mcm	140	99	P-3c1	165	28	P4_122	91	8	P-4	81	2
P2_1	4	94	P4_2/nmc	137	26	P4bm	100	8	Pnnn	48	2
R3m	160	88	I-42m	121	26	I2_12_12_1	24	7	Pnc2	30	2
P6_3	173	87	P-31m	162	23	Ibca	73	7	Pmmm	47	2
Pm-3m	221	87	R32	155	23	Iba2	45	7	P23	195	2
Cmce	64	86	Pmc2_1	26	22	Fmm2	42	7	I4_132	214	2

P2/c	13	82	P-6c2	188	22	P4_2/mbc	135	7	I-4c2	120	2
P4_2/mnm	136	76	P-43m	215	19	P4_132	213	7	Pm-3n	223	1
F-43m	216	75	Cmmm	65	18	P31m	157	7	P6_522	179	1
Pa-3	205	72	Pmma	51	17	P6_422	181	7	I4_1	80	1
Cc	9	71	Amm2	38	17	I4_1md	109	6	P312	149	1
I-4	82	71	Im-3m	229	16	I4cm	108	6	F222	22	1
P2_13	198	68	Pn-3	201	16	P6_1	169	6	Pm-3	200	1
P4/mmm	123	66	P-62c	190	15	P6/mmm	191	6	P4_2nm	102	1
Fddd	70	64	P4/mnc	128	15	Ccc2	37	6	Pban	50	1
Ibam	72	62	P31c	159	15	P4_2mc	105	6	F23	196	1
Pnnm	58	60	P4_2/n	86	15	I23	197	6	P6_4	172	1
Pbcm	57	58	Fmmm	69	14	I-4m2	119	6	P4_322	95	1
Pmn2_1	31	57	Aea2	41	14	Ccce	68	5	C222	21	1
Imma	74	57	P6_3/mcm	193	14	Fm-3	202	5	Pma2	28	1
I4_1/a	88	56	P4/n	85	14	Pnn2	34	5	P4/m	83	1
Cm	8	53	Imm2	44	14	P-42m	111	5	P3c1	158	1
Pbam	55	52	P2/m	10	13	Aem2	39	5			
P-43n	218	51	P-42_1c	114	13	I422	97	5			

[1] Phonon database at Kyoto University http://phonondb.mtl.kyoto-u.ac.jp/ (accessed 12/14/2022).
